# Editorial

**Published:** 1844-02-24

**Authors:** 


THE MEDICAL EXAMINER.

PHILADELPHIA, FEBRUARY 24, 1844.

A subscriber has sent us for insertion in the Examiner, a translation of some remarks on the evils resulting from the use of Pessaries, contained in the work on midwifery by Professor Moreau. We have not space for the paper at present, nor do we deem its publication necessary, inasmuch as the substance of it may be found in Churchhill and several other modern authors in the English language; and besides, it is not denied by any respectable physician of the present day, that much mischief may, and occasionally does, result from the employment of that instrument. The question, however, is not whether the *improper* use of the instrument may be productive of injury, but whether it may not be dispensed with altogether, at least in a great majority of the cases in which it is now employed by respectable practitioners. Nearly twenty years have elapsed since we have adopted the affirmative side of this question, and time and observation have only strengthened our conviction that the cases to which it is properly adapted are exceedingly rare.

Except when the prolapse of the uterus is so great that the organ falls without the sphincter vaginæ, or where there is recto or vesico-vaginal hernia, we can hardly conceive of a condition of the parts requiring the use of this mechanical means—a means most offensive to the moral feelings of the female, and at variance with cleanliness and personal comfort.

The following are the objections to the use of Pessaries, by Dr. Hamilton, long the distinguished Professor of midwifery, at Edinburgh. They are sufficiently intelligible "without note or comment."

"Firstly, They can only act as palliatives, whatever may be the degree of the disease.

"Secondly, They necessarily keep up a continued irritation in the passage, and of course excite a mucous discharge from the vagina.

"Thirdly, Unless properly adapted, they make injurious pressure on the contents of the pelvis.

"Fourthly, If not frequently taken out and cleansed, they become incrusted with a calcareous matter, which proves highly irritating.

"Fifthly, They subject the patient to the charge of a medical attendant for life.

"And lastly, Cases from time to time occur, where, from the laceration of the perineum, &c., no ordinary pessary can be retained."

### MEDICAL CLASSES.

In a former number we expressed the opinion that the number of Students in attendance at the different Medical Colleges, was unusually large the present season. Since then we have received printed Catalogues from several of the Institutions, from which it would appear that we were not mistaken in our estimate.

| | |
|---|---|
| That of the University of Pennsylvania numbers | 424 |
| Jefferson Medical College . . . | 341 |
| Albany Medical College . . . . | 108 |

From the other Colleges we have less precise information, but learn from various quarters that the Classes are large.

| | |
|---|---|
| At the Louisville Medical Institution the number is stated to be . . . . . | 243 |
| Lexington . . . . . . . | 212 |
| Cincinnati . . . . . . | 183 |
| Geneva . . . . . . . . | 150 |
| Boston . . . . . . , . | 150 |
| College of Physians and Surgeons, New York, . . . . . . | 182 |
| Kemper College, St. Louis, . . . . | 90 |

From the University College of New York, we have no information.

### NEW TEST FOR STRYCHNINE.

BY EUGENE MARCHAND.

It often happens that the chemist, when called upon to decide a medico-legal question, experiences some difficulty in proving the presence or nature of certain poisonous substances, either because he cannot procure a sufficiency of the poison, or else the reagents serving to characterise them are too little sensitive, or do not offer that degree of precision as to allow you to pronounce with certainty in a capital case. Among the organic alkalies thus far known, we are all aware that strychnine is the most poisonous. Hence the discovery of a reaction which enables you to detect with certainty very minimum quantities, becomes a desideratum.

I believe I have attained this object by the following process, which is so sensible as to give a yet very appreciable reaction, even when you operate upon an imponderable quantity of sulphate of strychnine.

When you triturate a very small portion of strychnine with a few drops of *concentrated sulphuric acid containing a hundredth of its weight of nitric acid*, the strychnine disappears without giving rise to any perceptible phenomenon: but if you add to the mixture merely an atom of *peroxide of lead*, it immediately developes a magnificent blue color, which rapidly passes to violet, then gradually to red, and finishes lastly, after several hours, by turning to canary yellow. This reaction is characteristic of strychnine, as it has been impossible for me, up to the present time, to find a substance which acts in a like manner, under similar circumstances.

When you act upon infinitely small quantities of strychnine, it is preferable, in order to render the reaction more sensible to triturate, in a dry state, a few particles of peroxide of lead with the organic akali, then to let fall upon the mixture a single drop of the acid liquid. You will then distinctly perceive, in a manner not to leave any doubt upon the mind, the series of coloration I have above described, even when acting upon a portion of strychnine imponderable, but approximately estimated at one thousandth of a grain.—*Am. Journ. Pharm. from Jour. de Pharm. et Chim.*

